# Subvalvular tissue mimicking valve detachment-like pathology by vertical aneurysm in Takayasu’s arteritis

**DOI:** 10.1093/icvts/ivab348

**Published:** 2021-12-15

**Authors:** Kentaro Kiryu, Itaru Igarashi, Takuya Wada, Hiroshi Yamamoto

**Affiliations:** Department of Cardiovascular Surgery, Akita University Graduate School of Medicine, Akita, Japan

**Keywords:** Takayasu’s arteritis, Sinus of Valsalva aneurysm, Prosthetic valve detachment

## Abstract

A 74-year-old woman with Takayasu’s arteritis previously underwent aortic valve replacement at 59 years old. She was initially diagnosed with aortic valve stenosis and valve detachment. Moreover, preoperative computed tomography revealed ∼40 mm distance between the coronary artery ostium and the prosthetic valve (PV) and an aneurysm at the sinus of Valsalva. A Bentall procedure was subsequently performed. Intraoperative findings revealed no detachment of the PV. Following PV removal, the subvalvular tissue was noted to protrude into the left ventricular outflow tract. Subsequently, it was revealed that the tissue could have interfered with the PV; however, the PV appeared to have been detached considering the imaging findings.

## INTRODUCTION

Takayasu’s arteritis (TA) is an inflammatory disease. When patients with TA develop aortic regurgitation, they are usually managed by aortic valve replacement (AVR). In TA, it was previously reported that the ascending aorta may remain an aneurysm after several years, even without overt dilatation [1, 2].

In this case, we encountered a patient whose imaging findings were suggestive of prosthetic valve (PV) detachment, with the patient presenting post-AVR, with TA.

## CASE REPORT

Akita University’s Ethics Committee does not require an application for approval of anonymized observational research. The patient provided informed consent.

A 74-year-old woman underwent AVR [with mechanical valve (19 mm)] and fistula closure for aortic regurgitation and left-coronary-artery-pulmonary-artery fistula at 59 years old. Her family had no significant history of cardiovascular disease. Intraoperative findings revealed an inflamed aortic wall, prompting a diagnosis of TA.

**Figure 1:**
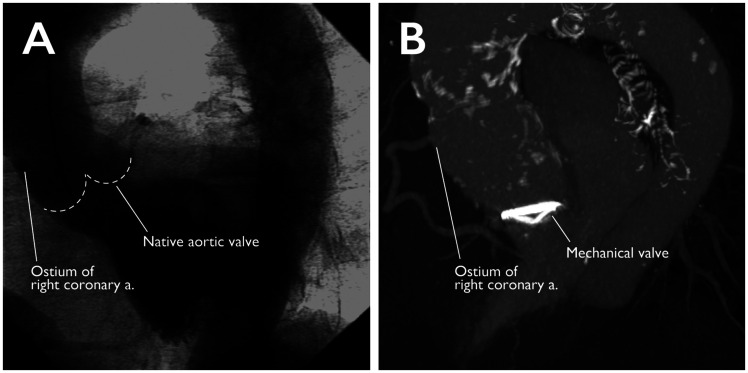
Morphological changes in the sinus of Valsalva. (**A**) The left ventriculogram before aortic valve replacement at age 59. The sinus of Valsalva, whose morphology is close to normal, can be observed. (**B**). A preoperative four-dimensional computed tomography at 74 years of age showing vertical dilation of the sinus of Valsalva; the height of the right coronary artery ostium remains changed from A and B (Th 8), indicating that the Valsalva's sinus is dilated in the left ventricular direction.

The patient complained of breathlessness on exertion since 1 month prior to presentation. Echocardiography and angiography revealed aortic stenosis (transaortic valve flow: 4.66 m/s, mean pressure gradient: 42 mmHg). Additionally, a restricted opening of the PV with the surrounding mosaic flow was observed (Video 1); detachment of the PV was suspected. However, the patient’s vital signs were stable, and blood tests subsequently showed no inflammatory response. Computed tomography and angiography revealed a sinus of Valsalva aneurysm (50 mm) and a distance between the coronary ostium and PV of ∼40 mm. This distance was significantly longer than that noted upon the patient’s AVR at 59 years old. Moreover, a caudal migration of the PV was observed; however, the right coronary ostium did not shift (Fig. 1 and Video 2). The Bentall procedure was subsequently performed.

**Figure 2:**
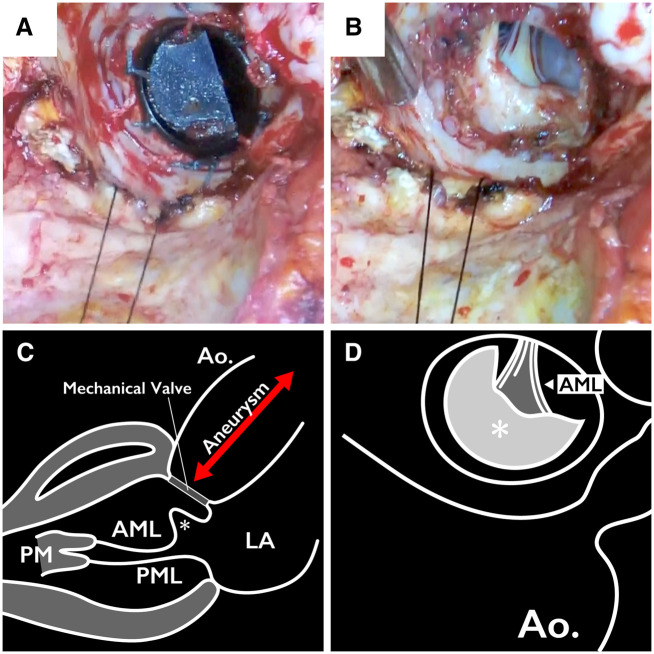
Intraoperative findings and schemas. (**A**) Before prosthetic valve removal. No features suggestive of prosthetic valve detachment. (**B**) Following prosthetic valve removal, a structure protruded under the prosthetic valve. (**C**) Schema illustrating the relationship between the aortic valve annulus and the vertically protruding structure (asterisk). (**D**) Schema of Figure B. The structure appears to protrude (asterisk) into the left ventricular outflow tract. AML, anterior mitral leaflet; PM, papillary muscle; PML, posterior mitral leaflet; LA, left atrium; Ao, aorta.

Intraoperatively, no detachment of the PV was observed (Fig. 2). Following PV removal, subvalvular tissue protruding into the left ventricular outflow tract (LVOT) was observed under the non-coronary cusp (Fig. 2). Because the subvalvular tissue intervened the aortic valve annulus and the anterior leaflet of the mitral valve, we considered it a mitral-aortic intervalvular fibrosa. Maximum excision of subvalvular tissue was performed to maintain the LVOT. To ensure the absence of pannus, we examined the removed PV, and it opened and closed smoothly.

On histopathology, mild lymphocyte infiltration into the aortic wall was observed, indicating prior inflammation.

On the 27th postoperative day, the patient developed sick sinus syndrome and underwent pacemaker implantation. On the 40th postoperative day, she was discharged and followed up by the cardiovascular medicine department.

## DISCUSSION

TA was described by Takayasu in 1908 [3]. TA causes inflammatory degeneration of arteries and can lead to aortic regurgitation. Sometimes, TA may require surgery; however, surgical interventions should be avoided in the active phase of the disease [4].

Anastomotic aneurysms after AVR have been reported to occur in 8.5% of TA patients [2]. Funada *et al.* [1] reported late presentation of an aneurysm from the sinus of Valsalva in a patient with TA who underwent AVR in only 6 reports, including theirs. A sinus of Valsalva aneurysm in TA shows diverse clinical features based on the direction of dilation. Especially, if it expands in the non-coronary cusp direction, it interferes with the atrioventricular node, resulting in an atrioventricular block [1]. Based on these findings, Matsuura *et al.* [5] recommended using the Bentall procedure from the beginning; however, AVR has been reported to have a better short-term prognosis.

In this case, intraoperative findings revealed an intact PV. A caudal shift of the PV was observed, showing vertical dilation of the sinus of Valsalva. These changes pushed the subvalvular tissue into the left ventricle (Figs 1 and 2), leading to protrusion into the LVOT, thus causing structural protrusion under the PV (Fig. 2). Furthermore, aberrant tissue interfered with the PV function, causing stenosis of the LVOT, and aortic stenosis was consequently considered.

This case demonstrated a rare detachment of the PV-like pathology, which was due to impaired PV function by a structure under the aortic valve annulus because of vertical dilation of the sinus of Valsalva. Such a rare presentation requires recognition. Therefore, following AVR in a patient with TA, strict follow-up is necessary.

**Conflict of interest:** none declared. 

## Reviewer information

Interactive CardioVascular and Thoracic Surgery thanks the anonymous reviewers for their contribution to the peer review process of this article.
